# A new network for the synergistic translation of mouse research

**DOI:** 10.1242/dmm.049865

**Published:** 2022-10-04

**Authors:** Owen Sansom

**Affiliations:** The Beatson Institute for Cancer Research, Glasgow University, Garscube Estate, Glasgow G61 1BD, UK

## Abstract

Over the past 20 years, the UK has become a leading force in the generation and use of complex mouse models in the precise investigation of human disease. Nevertheless, there remains a great challenge in improving how research in animals is translated to clinical benefits.

Developing and expanding connections between basic scientists and clinicians to ensure that animal models accurately recapitulate human disease will be key to this effort. This is the focus of the new UK Medical Research Council (MRC) National Mouse Genetics Network (https://nmgn.mrc.ukri.org/), which we believe will hugely impact our ability to harness recent advances in mouse genetics.

The National Mouse Genetics Network is a major £22 million investment initially comprising seven challenge-led research clusters with members distributed across the UK. At its core, the Mary Lyon Centre at MRC Harwell will act as a repository for, and provider of, genetically altered mice, as well as generate and share data, training, specialist facilities and resources. Importantly, each cluster will integrate expertise in fundamental biology with clinical findings to better address pertinent research questions.

Results from previous, smaller-scale, network initiatives suggest that this model can synergise research, but we believe that this structure will work better when carried out on a larger scale, with greater scope for collaboration and capacity of the system. This Editorial will outline the principal aims of the Network and identify the main areas in which this model will be able to exploit the power and synergy of its different elements.

## Teaser

**Summary:** This Editorial discusses a new initiative in pre-clinical mouse genetics research to increase the predictive power, relevance and sharing of complex genetic models by the UK MRC National Mouse Genetics Network.

## Cross-cluster synergy

We are excited to announce the first set of clusters to join the National Mouse Genetics Network and look forward to seeing how they will work together on a number of fantastic projects to develop the next generation of disease modelling in mice and to improve our ability to detect disease at the earliest stages of life. We are equally excited to see how others might join or interact with the Network, either by working with our existing clusters or by suggesting new research challenges around which to build future clusters.

**Figure:**
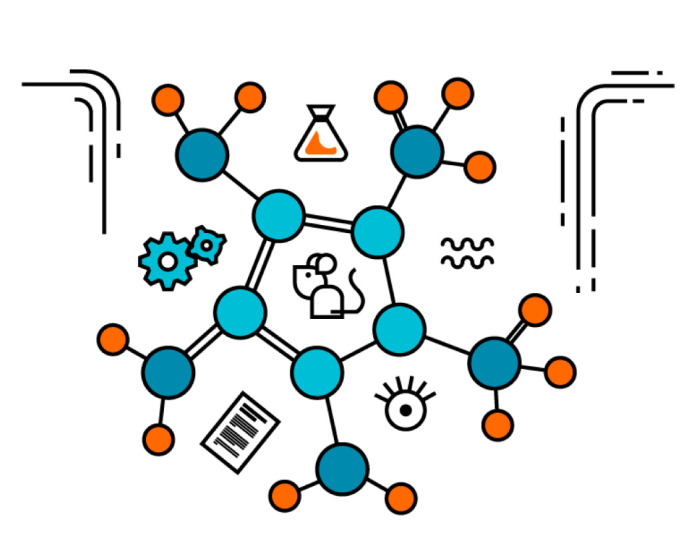
Image provided by the MRC National Mouse Genetics Network. For permission to reproduce, contact the MRC National Mouse Genetics Network.

Importantly, the Network will not just connect a linear series of technicians, scientists and clinicians with a shared disease interest. The clusters will be focused on distinctive, but complementary, research themes, with plans for cross-cluster and cross-disease interactions, while working on shared goals.

These shared objectives include areas that can broadly benefit animal research, such as the development or improvement of phenotyping pipelines for mouse models of disease, new and improved technologies for the generation of mouse mutants, and opportunities for alternatives to animal use. The Network will also contribute to highlighting shared biological pathways between different diseases and accelerate the discovery of therapeutic avenues.

Scientists can often become focused on their particular cellular system or organ of interest, but many diseases can be very wide-ranging in their effects, creating crossovers between otherwise very different disorders. Through creation of a wider network of experts across many disease areas, we can ensure that our understanding is as deep and broad as possible.

Our vision is that the Network will create a broad platform to improve access to mouse genetics for those working in translational medicine. This is aimed beyond our starting membership, as we believe that the Network will create a structure that is better placed than both individual institutes and smaller networks to engage with industry on the development of new disease therapeutics and to initiate new collaborations across academia.

## Capacity creation

A large network not only creates more points of contact to allow these kinds of interactions, but also creates greater capacity for a central organising hub. Indeed, one of the core strengths of the National Mouse Genetics Network will be its central hub, the Mary Lyon Centre.

We are in an age of big data, in which single-cell sequencing and deep molecular phenotyping will support the disease positioning of our mouse models so that they are as relevant as possible to human disorders. As well as acting as the repository for the mouse models themselves, the Mary Lyon Centre, along with a data platform initiative within the Network, will build the infrastructure required for open sharing of these data so that scientists within and outside of the Network can work together, efficiently, to improve our understanding of disease.

Our impact will also be accelerated through the new scientific and technical training centre, ADVANCE at MRC Harwell (https://har.mrc.ac.uk/training/). This centre strives to disseminate new methods, such as the use of home cage monitoring systems for behavioural studies and, in the near future, challenge-led phenotyping of neurological and metabolic disorders, as well as best practice in existing techniques, such as generation, validation and quality control of mouse models, efficient breeding and maintenance of mouse colonies, including germ-free strains for microbiome studies. All of these activities adhere to and promote the principles of the 3Rs (Replacement, Reduction and Refinement) that underpin our work and will benefit efficiency, reproducibility and translatability both within and outside of the Network, as well as expand the reach of our improved methods.

## The ability to integrate alternative models

The expansive contacts and interactions within and outside the Network will potentiate comparison and integration of data and experimental strategies from alternative models, allowing the replacement of some *in vivo* work and the optimisation of animal use. This underpins the firm commitment of the Network to lead the field of pre-clinical mouse genetics towards the full application of the 3Rs principles (Balls et al., 2009). For too long, there has been a focus on the single perfect model; instead, what is required is a suite of models, which are well positioned with human disease, and which can be used by the community at the appropriate time.

The Mary Lyon Centre has been at the forefront of this effort, with a demonstrable commitment to openness and an active outreach programme, ranging from engagement with national media to open days for local schools and interest groups. The Network will concentrate on the long-standing commitment of its members to best practices and will facilitate collaborations and joint projects between researchers making use of alternative models and those using mouse models, resulting in a more productive exchange between often insular fields of investigation.

## An opportunity to bridge the skills gap

The current job market has highlighted a significant shortage of skilled individuals willing to work in the field of pre-clinical genetics, as well as in many other areas of the science, technology, engineering and mathematics (STEM) subjects (https://assets.publishing.service.gov.uk/government/uploads/system/uploads/attachment_data/file/444048/High_level_STEM_skills_requirements_in_the_UK_labour_market_FINAL.pdf). The Network will be well positioned to identify the needs of scientists, clinicians and other Network-associated professionals and, making use of the Mary Lyon Centre's new ADVANCE training space, to devise a plan for the upskilling of existing Network-associated members and new staff. This will extend the reach of the wealth of expertise provided by the extensive web of professional knowledge within the Network. Such activity sits well within the remit specified by the MRC and will provide tangible benefits to the entire biomedical sciences community of the UK and beyond.

## Early interaction with industry

To be able to translate work from the laboratory to the clinic, interaction with industry is essential. One of the key concerns across all disease settings is that, historically, only a small percentage of pre-clinical studies translate to positive trials and licensed drugs. Furthermore, often pre-clinical researchers in academia only obtain small molecules and antibodies quite late in the development pipeline of a drug. Therefore, the path to translate exciting findings from the complex mouse models into clinical trials can be limited by the patent life or the stage of the development of that molecule. This can result in molecules being deprioritised by industry despite an excellent pre-clinical data package obtained from earlier results in other disease indications. Working in this context makes it hard for mouse geneticists to determine the predictive power of the models being studied. One of the objectives of the Network is to partner with industry early in the investigation of new drugs, and to use the robustness of our model systems to help determine the precise disease indication to be targeted. Our belief is that, by using more advanced models earlier in the translational pipeline, we can help develop more accurate and efficient therapeutics. Moreover, given the common mechanistic pathways that underpin many human diseases, such as the role of inflammation in diseases like cancer, obesity and heart disease, or the potential modulation of protein synthesis in infection, cancer and neurodegeneration (de Boer et al., 2020; Knight et al., 2020), we envisage that we will be able to support industry to interact with multiple disease indications.

## Filling the gap in data platforms and data-sharing capacity

One of our key aims is to share data with the national and international community, both within and outside the Network. Therefore, we plan to develop a data platform that will allow sharing of high-quality pre-clinical data. Importantly, one of our main objectives is to release data in a form that can be used by a broad range of researchers with varying levels of computational literacy. We also believe that there is a huge opportunity, given the recent work to profile human disease more deeply than ever before with single-cell and spatial approaches, to revolutionise the way in which we cross-compare human and mouse model data. This comes with the challenge of integrating large complex datasets. We believe it will also be important to provide the community with tools to achieve this key aim, and to provide access to, or distribute, these larger datasets in such a way that they are ready to use by computational experts.

## Conclusions

We hope that the Network will continue to grow and that we are at the start of a long-term strategy of investment. The purpose of the Network is to be fluid, to tackle key emerging questions and to identify synergies. We aim to help support funding applications, to stimulate interactions with the clusters and industry, and to provide opportunities for training and technology. There are many key problems in disease that we were not able to tackle in the first round of funding, and, by working with the mouse genetic community, we hope to develop further strategies and working groups to identify how we can synergise to address challenging questions, such as the impact of ageing and multimorbidity. To this end, we hope that interested investigators worldwide will sign up to be associates of the Network to help shape the future direction of our work.

The scope and overarching aims of the Network perfectly fit the founding principles of journals like Disease Models & Mechanisms (DMM) and could benefit from such journals supporting and promoting the Network's work and initiatives. The pillars of DMM are ‘quality disease research’ and ‘accessibility’. Central to our Network is the aim to build on excellent mechanistic science and support its translation to human disease through complex genetic models of disease. To achieve this, at the heart of the Network's strategy is sharing resources, data and best practice. By supporting cross-comparison of models within the community and aligning these models with human disease, we also hope to increase the robustness of pre-clinical modelling and understand variability. In this issue of DMM, the concept of cross-phylogenetic integration in human disease modelling is explored in more detail by Cheng et al. (2022) and also more specifically in the context of cancer modelling by Devlin and Roberts (2022). Furthermore, Svenson et al. (2022) provide an extensible discussion on recent National Institutes of Health (NIH) recommendations to improve rigor, translatability and transparency in pre-clinical studies, with a focus on mouse modelling. Journals, such as DMM, will help us accomplish our desired worldwide expansion and recruitment of associate members who could benefit from the Network and, at the same time, enhance its translational output.
